# Draft Genome Sequence of *Loktanella cinnabarina* Strain XM1 Isolated from Coastal Surface Water

**DOI:** 10.1128/genomeA.01310-17

**Published:** 2017-12-14

**Authors:** Ruijie Ma, Jianjun Wang, Qiong Wang, Zhaofeng Ma, Jia Li, Liqi Chen

**Affiliations:** a Institute of Marine Microbes and Ecospheres, State Key Laboratory of Marine Environmental Science, College of Ocean and Earth Sciences, Xiamen University, Xiamen, People’s Republic of China; b Key Laboratory of Global Change and Marine-Atmospheric Chemistry, Third Institute of Oceanography, State Oceanic Administration, Xiamen, People’s Republic of China; c Tianjin Branch of China National Offshore Oil Corporation Ltd., Tianjin, People’s Republic of China

## Abstract

We report here the draft genome sequence of *Loktanella cinnabarina* strain XM1, which was isolated from coastal surface water and shared 99.43% 16S rRNA gene sequence identity with the deep-sea bacterium *L. cinnabarina* LL-001^T^. The estimated genome size of strain XM1 is 3,782,785 bp, with a G+C content of 67.9%.

## GENOME ANNOUNCEMENT

The genus *Loktanella*, a member of the *Rhodobacter* group within the class *Alphaproteobacteria*, was first described by Van Trappen et al. in 2004 (1). To date, more than 20 draft *Loktanella* genomes have been sequenced and deposited in GenBank. Most members of this genus were isolated from freshwater and marine ecosystems, such as seawater (2–4), sea sand (5, 6), sediment (7), marine biofilms (8), and microbial mats in Antarctic lakes (1). In particular, *L. cinnabarina* LL-001^T^ was isolated from deep subseafloor sediment (9). Here, we report the draft genome sequence of *L. cinnabarina* strain XM1, which was isolated from a coastal surface water sample collected from Xiamen, China (24°30′N, 118°17′E), and has 99.43% 16S rRNA gene sequence identity with the deep-sea bacterium *L. cinnabarina* LL-001^T^. Colonies of *L. cinnabarina* strain XM1 are pink in color, while those of *L. cinnabarina* LL-001^T ^are vermilion.

Total genomic DNA of strain XM1 was extracted using the TIANamp bacteria DNA kit (Tiangen, Beijing, China). The draft genome sequencing of strain XM1 was performed at the Personal Biotechnology Co., Ltd. (Shanghai, China) with an Illumina MiSeq platform using a 2 × 301-bp paired-end DNA library. Sequencing resulted in a total of 3,039,150 reads and 752,681,545 nucleotide bases. Filtered reads were then assembled *de novo* using Newbler assembler version 2.8. GapCloser software was used to close gaps between the contigs and to generate final assembled sequences, which were then automatically annotated using the Rapid Annotations using Subsystems Technology (RAST) version 2.0 server (10).

The draft genome contains 92 contigs with a total length of 3,782,785 bp and a coverage depth of 89×. The largest contig is 585,710 bp, and the *N*_50_ contig length is 134,038 bp. Total genome length and average G+C content (67.9%) are consistent with those of other sequenced *Loktanella* isolates (8, 11). The coding region takes up 87.4% of the chromosome, and there are 4 rRNAs and 49 tRNAs identified in the genome of strain XM1. RAST analysis showed that the draft genome comprises 3,644 genes in total, 3,591 (98.5%) of which are protein-coding sequences (CDSs). Among them, 3,037 (84.6%) are assigned to biological functional proteins, while the remaining genes are annotated as hypothetical proteins. The abundant subsystems of protein-coding sequences are most related to the metabolic capacity of substrates, including carbohydrates (10.7%); amino acids and derivatives (10.2%); cofactors, vitamins, prosthetic groups, and pigments (8.3%); proteins (6.6%); and membrane transporters (4.3%). Although the 16S rRNA gene sequence of strain XM1 is more similar to *L. cinnabarina* strain LL-001^T^, a higher average nucleotide identity (ANI) value is observed between strain XM1 and *L. hongkongensis* DSM 17492^T^ (86.88%) than between strain XM1 and *L. cinnabarina* strain LL-001^T^ (86.12%), as determined by the online ANI calculator (12). It is noteworthy that colonies of strain *L. hongkongensis* DSM 17492^T^, isolated from a marine biofilm in Hong Kong waters (8), have the same pink color as those of strain XM1. With the increasing amount of genome information about *Loktanella* species isolated from different environments, we believe that further analysis will shed new light on the lifestyle and evolution of this widely distributed genus.

### Accession number(s).

This whole-genome shotgun project has been deposited at DDBJ/ENA/GenBank under accession number NQWH00000000. The version described in this paper is the first version, NQWH01000000.
